# Does puberty moderate the relationship between personality and eating disorders?

**DOI:** 10.1186/2050-2974-3-S1-O26

**Published:** 2015-11-23

**Authors:** Chiara Paganini, Gregory Peterson, Matthew Fuller-Tyszkiewicz, Marija Anderluh, David Collier, Fernando Fernandez-Aranda, Andreas Karwautz, Gudrun Wagner, Nadia Micali, Janet Treasure, Isabel Krug

**Affiliations:** 1 University of Tasmania, Australia; 2 Deakin University, Australia; 3 University of Ljubljana, Slovenia; 4 King's College, United Kingdon; 5 University of Barcelona, Spain; 6 University of Vienna, Austria; 7 University College London, UK; 8 University of Melbourne, Australia

## Aim

To investigate the moderating effect of timing and attitudes towards puberty on personality traits and severity of eating disorder (ED) symptomatology in a clinical ED sample and to assess whether differences existed between Anorexia Nervosa (AN) and Bulimia Nervosa (BN) patients.

## Method

354 ED patients [AN-Restrictive (AN-R) =109, AN-Binge Purging A(n-BP) =168 and BN=77]. Pubertal risk factors were assessed through the ORFI and the EATATE. ED symptom severity was assessed through the EDI-2 and personality through the TCI-R temperament scales: Novelty Seeking, Harm Avoidance, Reward Dependency and Persistence.

## Result

A range of pubertal risk factors significantly moderated the relationship between temperaments and ED severity. In particular, the relationship between TCI-Novelty Seeking and ED severity was found to be positively moderated by a negative attitude toward menstruation (p<0.01) and negatively moderated by earlier breast development (p<0.05). Similarly, the relationships between TCI-Harm Avoidance, TCI-Reward Dependency and TCI-Persistence and ED severity were found to be positively moderated by an earlier age of menarche and negatively moderated by a negative attitude towards breast development (p<0.05). Regarding ED subtypes, the only significant findings were obtained for AN-R patients.

## Conclusion

These findings suggest that puberty moderates temperaments and consequent ED severity. The assessment of personality provides important insights into which girls struggle more with pubertal development and are at increased risk of EDs.

